# TNF Induction of NF-κB RelB Enhances RANKL-Induced Osteoclastogenesis by Promoting Inflammatory Macrophage Differentiation but also Limits It through Suppression of NFATc1 Expression

**DOI:** 10.1371/journal.pone.0135728

**Published:** 2015-08-19

**Authors:** Zhijun Zhao, Xiaodong Hou, Xiaoxiang Yin, Yanyun Li, Rong Duan, Brendan F. Boyce, Zhenqiang Yao

**Affiliations:** 1 Department of Medical Imaging, Henan University First Affiliated Hospital, 357 Ximen Street, Kaifeng, Henan 475001, P.R. China; 2 University of Rochester Medical Center, Department of Pathology and Laboratory Medicine and Center for Musculoskeletal Research, Box 626, Room 1–2105, 601 Elmwood Ave, Rochester, NY 14642, United States of America; University of Oulu, FINLAND

## Abstract

TNF induces bone loss in common bone diseases by promoting osteoclast formation directly and indirectly, but it also limits osteoclast formation by inducing expression of NF-κB p100. Osteoclast precursors (OCPs) are derived from M1 (inflammatory) and M2 (resident) macrophages. However, it is not known if TNF stimulates or limits osteoclast formation through regulation of M1 or M2 differentiation or if RelB, a partner of p100, is involved. To investigate these questions, we treated bone marrow cells (BMCs) with M-CSF alone or in combination with TNF to enrich for OCPs, which we called M-OCPs and T-OCPs, respectively. We found that TNF switched CD11b^+^F4/80^+^ M-OCPs from Ly6C^-^Gr1^-^ M2 to Ly6C^+^Gr1^-^CD11c^+^ and Ly6C^-^Gr1^-^CD11c^+^ M1 cells. RANKL induced osteoclast formation from both Ly6C^+^Gr1^-^ and Ly6C^-^Gr1^-^ T-OCPs, but only from Ly6C^+^Gr1^-^ M-OCPs, which formed significantly fewer osteoclasts than T-OCPs. Importantly, Ly6C^+^Gr1^-^ cells from both M- and T-OCPs have increased expression of the M1 marker genes, iNOS, TNF, IL-1β and TGFβ1, compared to Ly6C^-^Gr1^-^ cells, and Ly6C^-^Gr1^-^ cells from T-OCPs also have increased expression of iNOS and TGFβ1 compared to cells from M-OCPs. Both RANKL and TNF increased RelB mRNA expression. TNF significantly increased RelB protein levels, but RANKL did not because it also induced RelB proteasomal degradation. TNF inhibited RANKL-induced NFATc1 mRNA expression and osteoclast formation from M-OCPs, but not from T-OCPs, and it did not induce Ly6C^+^Gr1^-^CD11c^+^ or Ly6C^-^Gr1^-^CD11c^+^ M1 macrophages from RelB-/- BMCs. Furthermore, overexpression of RelB in M-OCPs reduced RANKL-induced osteoclast formation and NFATc1 mRNA expression, but it increased TNF-induced OC formation without affecting NFATc1 levels. Thus, TNF induction of RelB directly mediates terminal osteoclast differentiation independent of NFATc1 and limits RANKL-induced osteoclastogenesis by inhibiting NFATc1 activation. However, the dominant role of TNF is to expand the OCP pool by switching the differentiation of M-CSF-induced M2 to M1 macrophages with enhanced osteoclast forming potential. Strategies to degrade RelB could prevent TNF-induced M2/M1 switching and reduce osteoclast formation.

## Introduction

TNF is the major cytokine driving inflammation in rheumatoid arthritis (RA), a chronic inflammatory disease affecting about 1% of the world's population and characterized by synovial inflammation and joint destruction, leading to severe morbidity and premature mortality [1]. Transgenic mice over-expressing TNF (TNF-Tg mice) develop a form of arthritis that is very similar to human RA [2]. Although anti-TNF therapies have significantly reduced the morbidity and joint destruction in RA, they are expensive, and only about 60% of patients have a good response to these agents [3, 4]. In non-responding patients, TNF inhibitors typically are administered for several months before a decision is made to switch to an alternative treatment, which is often another TNF inhibitor that also may be ineffective. Thus, there is a need to better understand how TNF induces joint inflammation and destruction.

Inflammatory cells, such as lymphocytes, macrophages and mast cells, drive chronic inflammatory processes, including synovial inflammation, by producing cytokines and autoantibodies at involved sites. Joint destruction in RA is mediated by ectopic differentiation of osteoclasts (OCs) from their monocyte-macrophage lineage precursors in affected joints. Receptor activator of nuclear factor-κB ligand (RANKL), a member of the TNF superfamily, mainly controls later phases of OC differentiation and activation [5], and its expression by synoviocytes and inflammatory cells in affected joints is promoted by TNF and other cytokines [6, 7]. RANKL expression is also required for normal B cell development and lymph node formation [8], suggesting that it might have a role to promote joint inflammation in RA. However, TNF-Tg mice generated to have deficiency of RANKL also develop synovial inflammation, but not joint destruction because OCs do not form in these mice [9, 10]. Preclinical and clinical studies indicate that RANKL inhibitors do not significantly alter inflammatory processes in RA [11]. These findings suggest that RANKL does not contribute significantly to TNF-induced inflammation in RA.

TNF can induce osteoclastogenesis directly from *Rank* ^–/–^OC precursors (OCPs) in vitro when the cells are co-cultured with [12] or without [13] TGF-β1, which is released from bone matrix during bone resorption and activated by the acidic microenvironment in resorption lacunae as a result of acid release from OCs [14, 15]. However, the numbers of OCs induced by TNF from WT OCPs are much lower than those induced by RANKL [16]. Despite these findings, it was puzzling that TNF did not induce OC formation when administered in vivo to *Rank* ^–/–^mice [17]. We have reported that TNF induces expression of NF-κB p100 and that p100 limits TNF- and RANKL-mediated OC formation [13]. Consistent with this inhibitory effect of p100, we also found that TNF efficiently induced OC formation in vivo when it was administered to RANKL^-/-^ or RANK^-/-^ mice also deficient in p100 [13]. TNF-Tg mice that we generated to be deficient in p100 have significantly accelerated development of arthritis and systemic bone loss, suggesting that p100 not only limits OC formation, but also joint inflammation induced by TNF [13]. More recently, it was reported that TNF also limits OC formation through RBP-j [18] and IRF-8 [19], indicating that there are several mechanisms to restrict the destructive effects of TNF on bone. In contrast, TNF can also synergize with RANKL to induce OC formation [20, 21]. However, the precise conditions in which TNF limits or promotes OC formation and the factors that are critical for TNF induction or inhibition of OC formation remain unclear.

OCPs comprise both classically activated (inflammatory, M1) macrophages and alternatively activated (resident, M2) macrophages [22–24]. LPS, which induces TNF production [25], promotes the differentiation of M1 macrophages [22, 24], and TNF increases the numbers of circulating OCPs by promoting their proliferation through up-regulation of expression of the receptor for M-CSF [26]. Expression of M-CSF, like RANKL, is essential for OC formation [27]. However, it is not known if TNF induction or inhibition of OC formation involves modulation of M1/M2 macrophage differentiation into OCs. We report here that TNF switches the differentiation of M-CSF-primed M2 into M1 macrophages to enhance osteoclastogenesis by inducing the expression of NF-κB RelB, the partner of p100/p52, and that RelB also directly targets NFATc1 to limit OC formation.

## Materials and Methods

### Reagents

Recombinant murine M-CSF, RANKL, TNF were purchased from R&D Systems (Minneapolis, MN). Antibodies of RelB for Western blot were purchased from Santa Cruz and anti-actin antibody was from Sigma. Goat anti-rabbit (or mouse) IgG-HRP conjugate secondary antibody was purchased from Bio-Rad. Fluorescent-labeled rat anti-mouse antibodies APC-Ly6C, PEcy7-CD11b, PE-Gr1 (also called Ly6G) and PECy5-F4/80 were purchased from eBioscience. Ammonium Chloride (NH4Cl) solution was purchased from STEMCELL technologies.

### Animals

Cells from 6–8 week-old C57Bl6 mice as well as RelB-/- and WT littermate mice on an inbred C57BL/6 background [28–30] were used for in vitro studies. This study was carried out in strict accordance with the administrative regulations of Laboratory Animals of the National Science and Technology Commission of People’s Republic of China and the recommendations in the Guide for the Care and Use of Laboratory Animals of the National Institutes of Health. The protocols were approved by the Committee on the Ethics of Animal Experiments of Henan University and the University of Rochester Medical Center Institutional Animal Care and Use Committee. Mice were euthanized by CO_2_ followed by neck dislocation according to AVMA guidelines.

### Osteoclastogenesis

The culture procedure was modified from our previous reports [13, 16, 26, 31]. Briefly, bone marrow (BM) was flushed from the long bones of mice using α-MEM containing 10% FBS, passed through a 21-G and then through a 25-G needle to make single cell suspensions. The cells were incubated in NH4Cl solution for 10 min at room temperature to lyse red blood cells. 6x10^4^ BM cells were seeded in 96-well plates and cultured with 5 ng/ml M-CSF for 2 days, and then RANKL (10 ng/ml) or TNF (20 ng/ml) or both were added to the cultures for 48–56 hr at which time mature OCs could be observed using an inverted microscope. The cells were then fixed with 10% neutral phosphate-buffered formalin for 10 min and TRAP staining was performed. TRAP+ cells with three or more nuclei were considered as mature OCs.

### Over-expression of RelB

BM cells prepared as above were seeded in 96-well plates (4x10^4^/well) for the evaluation of OC formation or in 60-mm dishes (1.2x10^6^ cells per dish) for Western blot or FACS analysis followed by culture with M-CSF for 2 days to enrich for OCPs. For over-expression of RelB, the culture medium was replaced with fresh medium containing M-CSF, 2 μg/ml polybrene and ¼ volume of retro-viral supernatant of pMX-GFP or pMX-GFP-RelB prepared from Plat-E packaging cells, as we described previously [13, 16, 31]. After 24 hr of infection, RANKL or TNF was added to the cultures for an additional 48–96 hr to generate OCs.

### FACS analysis and cell sorting

2×10^6^ BM or cultured cells were stained with APC-Ly6C, PEcy7-CD11b, PE-Gr1 and PECy5-F4/80 antibodies for 30 min. Data were acquired using a FACScanto flow cytometer and analyzed using FlowJo software, as described previously [26, 30]. For cell sorting, cultured OCPs from WT mice were stained with the above fluorescent-conjugated antibodies to confirm that each cell population was similar to those generated in our regular culture procedure, and the cells were then sorted by a Statler sorter to collect Ly6C^+^Gr1^-^, Ly6C^+^Gr1^+^, Ly6C^-^Gr1^-^ and Ly6C^-^Gr1^+^ cells separately. The sorted cells were reanalyzed to ensure their purity (≥98%) and used for osteoclastogenesis assays, as we described previously [26].

### Western blot analysis

Cultured cells were lysed with M-Per mammalian protein extraction reagent (Thermo Scientific) containing a protease inhibitor cocktail (Sigma). Lysate proteins (10–20 μg) were loaded in 10% SDS-PAGE gels and transferred onto polyvinylidene difluoride membranes. Following blocking in 5% milk, membranes were incubated with a specific primary antibody to RelB or mouse β-actin over-night at 4°C. After washing, the membranes were incubated with anti-rabbit (or mouse) IgG-HRP conjugate secondary antibody (Bio-Rad) and exposed to ECL substrate. Signals were analyzed using a Bio-Rad imaging system.

### Quantitative Real-Time PCR

Total RNA was extracted from cultured cells using 1 ml TRIzol reagent, and 1 μg of RNA was used for synthesis of cDNA using a GeneAmp RNA PCR core kit. Quantitative PCR amplification was performed using an iCycler real-time PCR machine and iQ SYBR Green (Bio-Rad). Relative mRNA expression levels of target genes were analyzed using the CT value of the gene, normalized to β-actin.

### Statistics

All results are given as the mean ± S.D. Comparisons between two groups were analyzed using Student's two-tailed unpaired t test. One-way analysis of variance and Dunnett's post hoc multiple comparisons were used for comparisons among three or more groups. p values <0.05 were considered statistically significant. Each experiment was repeated at least twice with similar results.

## Results

### TNF switches the differentiation of M-CSF-induced Ly6C^-^Gr1^-^ M2 to Ly6C^+^Gr1^-^CD11c^+^ and Ly6C^-^Gr1^-^CD11c^+^ inflammatory M1 macrophages

OCPs belong to the monocyte/macrophage lineage. Mouse CD11b^+^Gr1^-/lo^ cells are precursors that efficiently form OCs [26]. Since macrophages have been classified as M1 and M2 cells and TNF generally stimulates M1 differentiation [23], we investigated if TNF regulates M1/M2 cells and if this affects their OC formation potential. More than 30 cell surface markers are used to classify M1 and M2 macrophages [22, 23]. CD11b and F4/80 are common macrophage surface markers; M1 cells are CCR2^+^Gr1^+^CD62L^+^CD11c^+^, while M2 cells are CCR2^-^Gr1^-^CD62L^-^CD11c^-^ in mice [22, 23]. Ly6C is also widely used to differentiate M1 and M2 cells. Ly6C^+/hi^ subsets are classified as M1 cells, while Ly6C^-/lo^ subsets are classified as M2 cells [32, 33].

**Fig 1:**
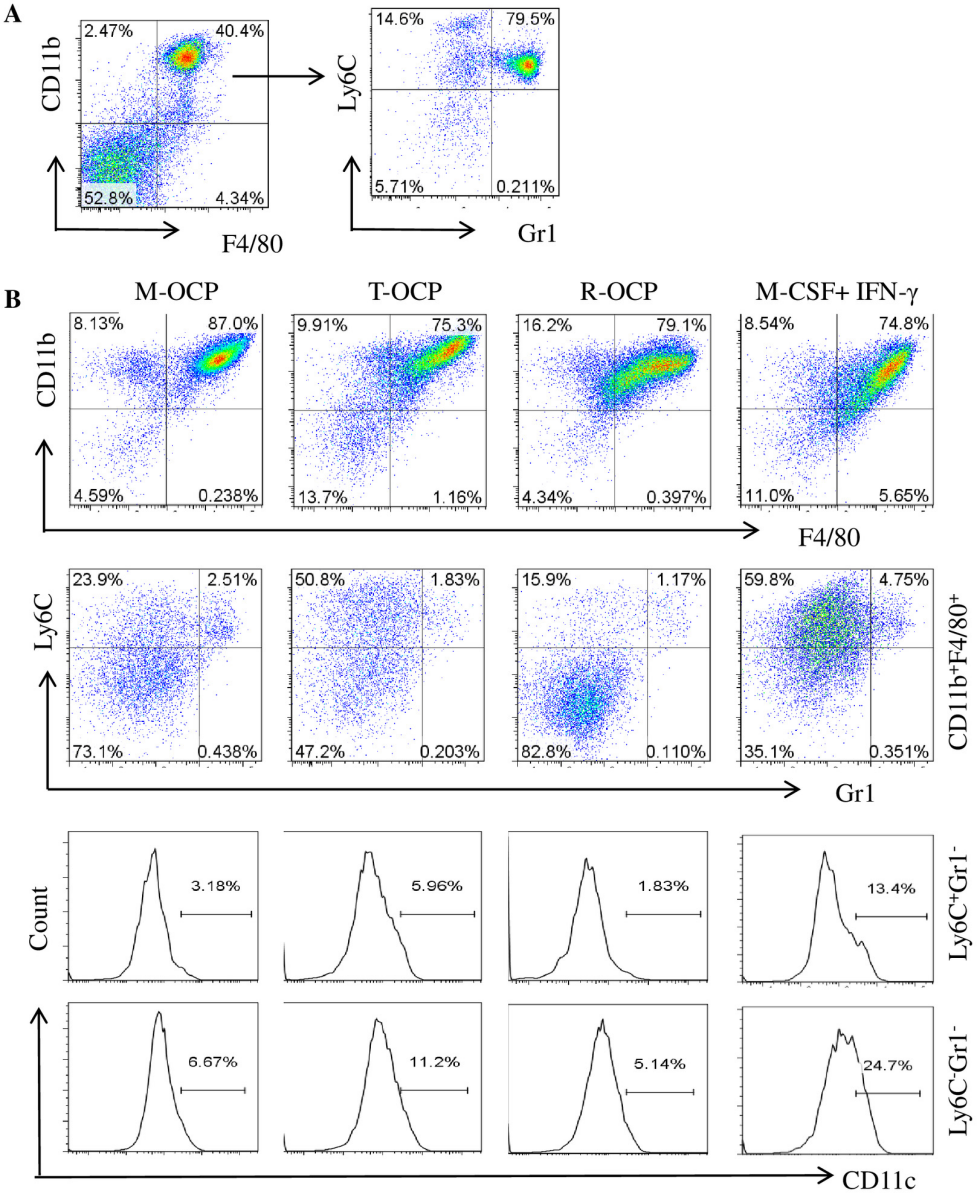
TNF promotes the differentiation of Ly6C^+^Gr1^-^CD11c^+^ M1 macrophages. (A) Freshly isolated bone marrow cells (BMCs) from 3-month-old C57Bl6 mice were stained with anti-mouse APC-Ly6C, PEcy7-CD11b, PE-Gr1, FITC-CD11c and PEcy5-F4/80 antibodies and expression levels of these cell surface markers were analyzed by flow cytometry. (B) BMCs (2x10^6^) from the mice in (A) were cultured with M-CSF, M-CSF+TNF (20ng/ml) or M-CSF+RANKL (10ng/ml) in 60 mm-dishes for 3 days to recruit OCPs, which we called M-CSF-induced OCPs (M-OCPs), TNF-induced OCPs (T-OCPs), and RANKL-induced OCPs (R-OCPs), respectively. IFN-γ (1ng/ml) was also added to M-CSF-treated cells as a positive control for M1 macrophage recruitment. Cells attached to the dishes were collected and stained with the above antibodies to analyze expression of cell surface markers by flow cytometry: CD11b^+^F4/80^+^ cells in the total cultured OCPs (upper panel), Ly6C^+^Gr1^-^ and Ly6C^-^Gr1^-^ cells in the CD11b^+^F4/80^+^ population (middle panel) and CD11c^+^ cells in the Ly6C^+^Gr1^-^ and Ly6C^-^Gr1^-^ populations (lower panel). The experiment was repeated three times with similar results.

To characterize TNF-induced OCPs, we examined expression of the cell surface markers: CD11b, F4/80, Ly6C, CD11c and Gr1. About 40% of primary BM cells were CD11b^+^F4/80^+^ cells and of these ~80% were Ly6C^+^Gr1^hi+^ granulocytes and ~15% were Ly6C^+^Gr1^-/lo+^ cells (Fig 1A), corresponding to our previously reported CD11b^+^Gr1^-/lo+^ OCPs [26]. We cultured BM cells with M-CSF alone or in combination with TNF or RANKL for 3 days to generate OCPs, which we called M-CSF-induced OCPs (M-OCPs), TNF-induced OCPs (T-OCPs) and RANKL-induced OCPs (R-OCPs), respectively. We found that the two common macrophage surface markers, CD11b^+^F4/80^+^, comprised ~75% and ~79% of T- and R-OCPs respectively, compared to ~87% of M-OCPs (p<0.05, Fig 1B upper panel and S1 Fig). We also found that in these CD11b^+^F4/80^+^ populations, Ly6C^+^Gr1^-^ cells comprised 23.9%, 50.8% and 15.9% of M-OCPs, T-OCPs and R-OCPs, respectively, while Ly6C^-^Gr1^-^ cells comprised 73%, 47% and 82.8% of these cells, respectively (Fig 1B middle panel), suggesting that TNF switched M-CSF-induced Ly6C^-^Gr1^-^ M2 to Ly6C^+^Gr1^-^ M1 macrophages, an effect similar to that induced by IFN-γ, which is a standard stimulator of M1 macrophage differentiation [22, 23]. In contrast, RANKL did not change the phenotype of M-CSF-induced macrophages. We also analyzed another M1 marker, CD11c, in Ly6C^+^Gr1^-^ and Ly6C^-^Gr1^-^ cells, and found that in the Ly6C^+^Gr1^-^ cells from the CD11b^+^F4/80^+^ populations, the frequency of CD11c^+^ cells in T-OCPs (5.96%) was double that in M-OCPs (3.18%), while it remained low in R-OCPs (1.83%, Fig 1B lowest panel). Similarly, in the Ly6C^-^Gr1^-^ cells from the CD11b^+^F4/80^+^ populations, CD11c^+^ cells in T-OCPs (11.2%) were almost twice as frequent as those that in M-OCPs (6.67%) (Fig 1B lowest panel). We used IFN-γ as a positive control and found that it also increased CD11c^+^ cells in both Ly6C^+^Gr1^-^ and Ly6C^-^Gr1^-^ cells from CD11b^+^F4/80^+^ populations, about four-fold more than those that in M-OCPs (Fig 1B lowest panel). These data suggest that Ly6C^-^Gr1^-^ cells from T-OCPs had also shifted to M1 macrophages.

### TNF-induced CD11b^+^F4/80^+^Ly6C^+^Gr1^-^ and CD11b^+^F4/80^+^Ly6C^-^Gr1^-^CD11c^+^ macrophages have higher OC forming potential than M-CSF-induced macrophages

**Fig 2:**
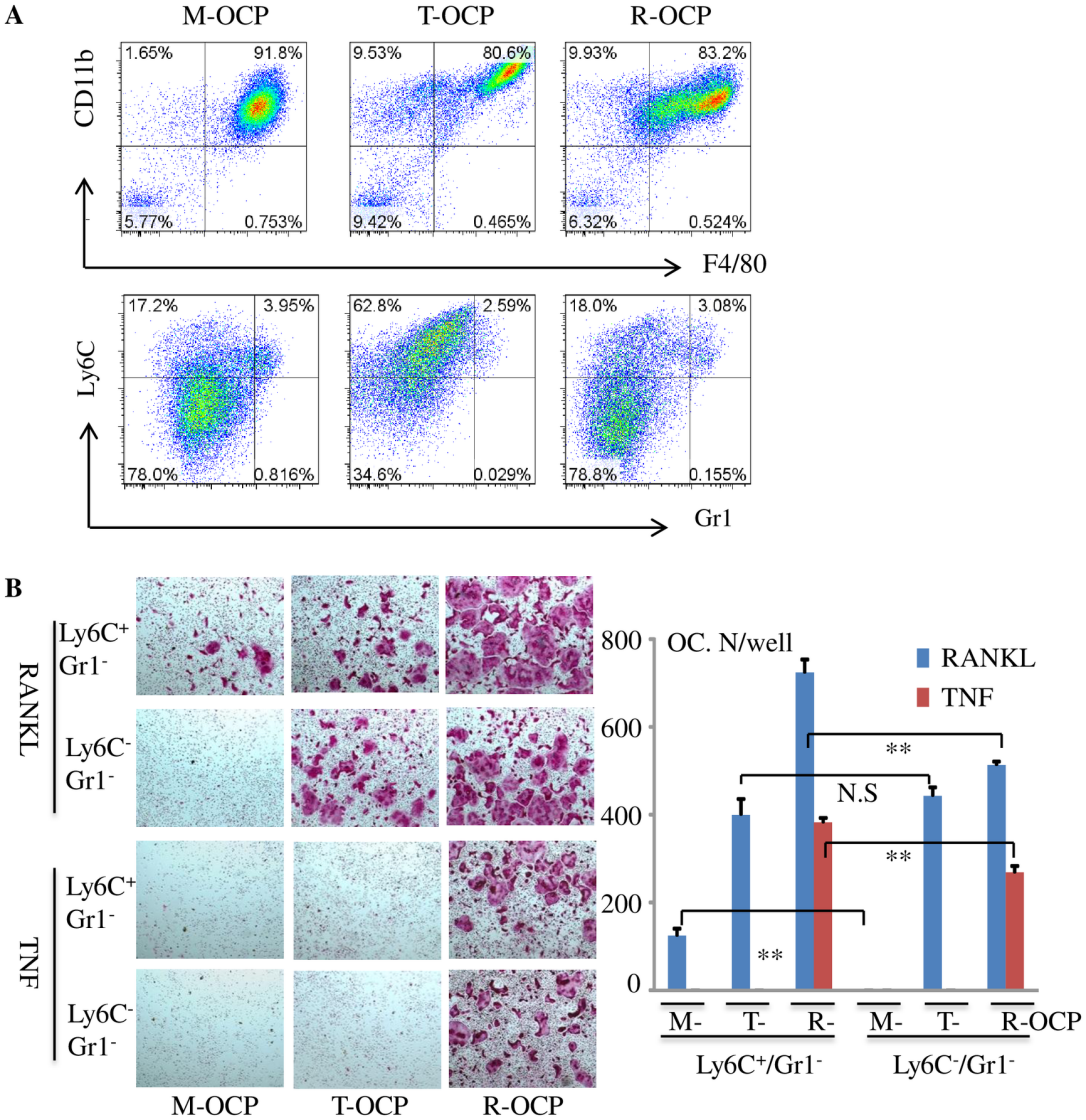
TNF-induced macrophages have higher OC forming potential than M-CSF-induced macrophages. (A) M-, T-, and R-OCPs cultured from BMCs from a 4-month-old C57Bl6 mouse were stained with the fluorescent-labeled antibodies as in Fig 1. Ly6C^+^Gr1^-^ and Ly6C^-^Gr1^-^ populations from CD11b^**+**^F4/80^**+**^ cells were sorted by flow cytometry. (B) The sorted cell populations were seeded in 96-well plates (4x10^4^ cells/well) and treated with RANKL or TNF in the presence of M-CSF for 2 additional days to generate mature OCs, which were stained for TRAP activity. (C) Quantitation of numbers of OCs formed from each sorted population in (B), 4 wells per group, *p < 0.05, **p < 0.01. The experiment was repeated twice with similar results. M = M-CSF, P = PBS, R = RANKL, T = TNF, R+T = RANKL+TNF.

M-, T-, and R-OCPs generated from WT mouse BM cells were stained with PEcy7-CD11b, PEcy5-F4/80, APC-Ly6C and PE-Gr1 antibodies to analyze their macrophage surface markers. We confirmed that the expression levels of cell surface markers in each OCP type (Fig 2A upper panel) were similar to those in OCPs in Fig 1B. We sorted each of these OCPs into four populations: Ly6C^+^Gr1^-^, Ly6C^+^Gr1^+^, Ly6C^-^Gr1^-^ and Ly6C^-^Gr1^+^ cells. 4x10^4^ of the sorted cells were seeded into 96-well plates and treated with RANKL or TNF in the presence of M-CSF to induce OC formation. In M-OCPs, the OC forming potential of Ly6C^+^Gr1^-^ cells was much higher than Ly6C^-^Gr1^-^ cells: RANKL induced 125±16 OCs from Ly6C^+^Gr1^-^ cells, but only a few TRAP+ mononuclear cells were formed from Ly6C^-^Gr1^-^ cells at this time point (Fig 2B). In contrast, both Ly6C^+^Gr1^-^ and Ly6C^-^Gr1^-^ cells from T- and R-OCPs formed many OCs, and both Ly6C^+^Gr1^-^ and Ly6C^-^Gr1^-^ cells from T-OCPs formed similar numbers of OCs, but Ly6C^+^Gr1^-^ cells from R-OCPs formed more OCs than Ly6C^-^Gr1^-^ cells in response to RANKL (p<0.01). In addition, OC numbers from Ly6C^-^Gr1^-^ T-OCPs almost matched those from R-OCPs. TNF also induced large numbers of OCs from Ly6C^+^Gr1^-^ and Ly6C^-^Gr1^-^ cells from R-OCPs (Fig 2B) probably because R-OCPs had already undergone some further differentiation. There were fewer Gr1^+^ cells (Ly6C^+^Gr1^+^and Ly6C^-^Gr1^+^) in these cultured OCPs (Fig 2A) and the Gr1^+^ cells from M-OCPs did not form OCs in response to TNF or RANKL (not shown). RANKL also induced OC formation from Ly6C^+^Gr1^+^ cells from T- and R-OCPs (not shown), but the total numbers of these cells were small.

### Ly6C^+^Gr1^-^ cells express M1 macrophage markers and Ly6C^-^Gr1^-^ cells from T-OCPs are also polarized to M1 macrophages

**Fig 3:**
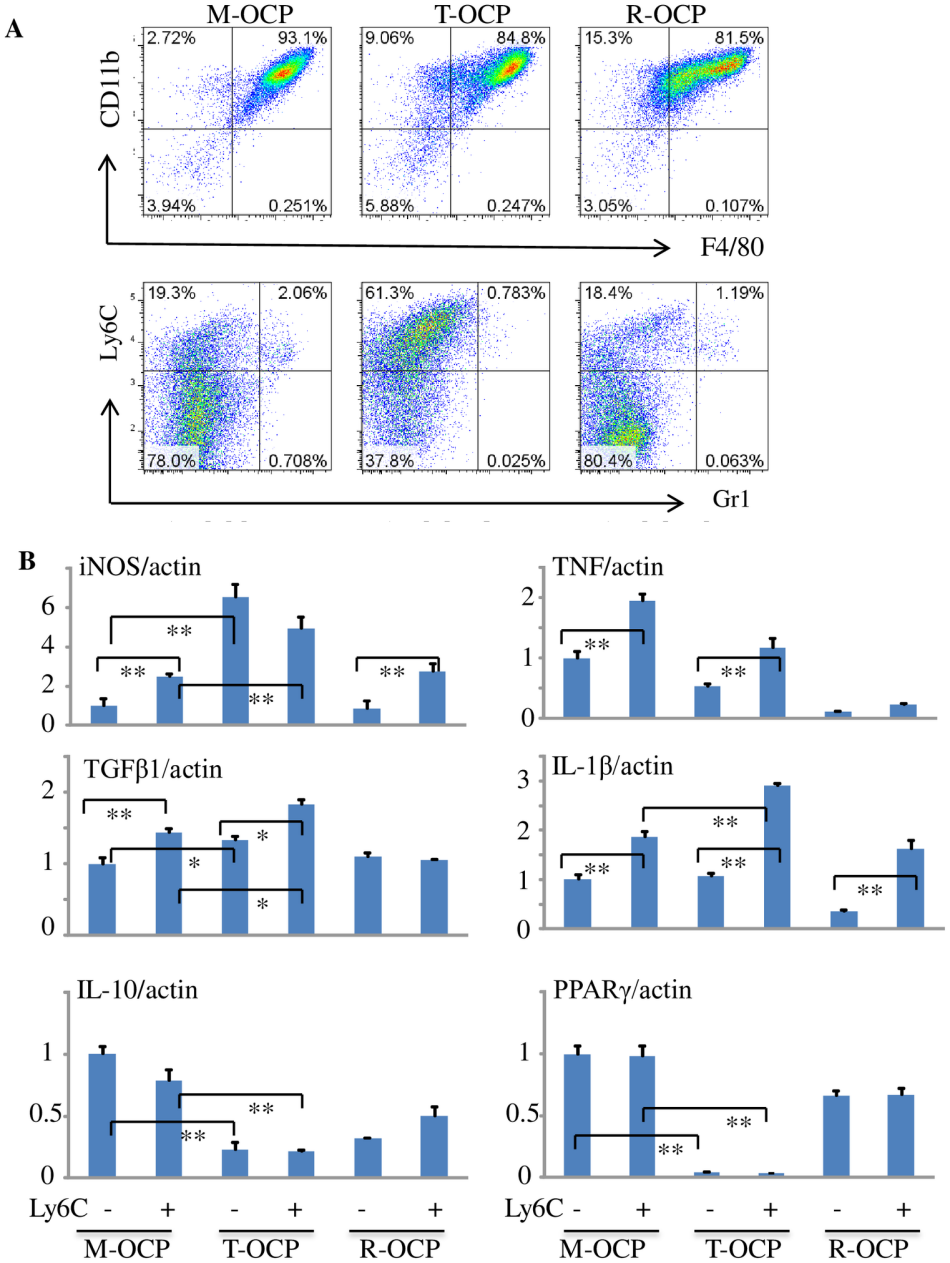
TNF-induced macrophages express M1 markers. Ly6C^+^Gr1^-^ and Ly6C^-^Gr1^-^ cells were sorted from cultured M-, T- and R-OCPs from 3-month-old C57Bl6 mice, as in Fig 2A (A). Total RNA was extracted from these sorted cells and mRNA expression levels of the M1 macrophage genes, TNF-α, iNOS, IL-1β and TGFβ1 as well as the M2 macrophage marker genes, PPAR-γ and IL-10, were tested by real-time PCR, normalized to β-actin (B). The data are representative of two independent experiments. *p < 0.05, **p < 0.01.

We next sorted Ly6C^+^Gr1^-^ and Ly6C^-^Gr1^-^ cells from M-, T- and R-OCPs (Fig 3A) to extract total RNA. We used 1 μg RNA from each sample to reverse transcribe cDNA to test levels of the M1 marker genes, iNOS, TNF, TGFβ1 and IL-1β as well as the M2 markers, IL-10 and PPAR-γ, by real-time PCR. We found that the expression levels of iNOS, TNF, TGFβ1 and IL-1β were increased by 2.5, 1.95, 1.62 and 1.87 fold (p<0.05), respectively, in Ly6C^+^Gr1^-^ cells from M-OCPs compared to Ly6C^-^Gr1^-^ cells, while the levels of IL-10 and PPAR-γ were not significantly different (Fig 3B), confirming that Ly6C^+^Gr1^-^ cells generally have a M1 profile. Ly6C^+^Gr1^-^ cells from T-OCPs expressed higher iNOS, IL-1β and TGFβ1, but significantly lower IL-10 and PPAR-γ levels than those from M-OCPs (p<0.05). Of note, expression levels of iNOS and TGFβ1 were also increased, while IL-10 and PPAR-γ levels were decreased in Ly6C^-^Gr1^-^ cells from T-OCPs compared to M-OCPs cells (Fig 3B), providing further support that Ly6C^-^Gr1^-^ cells from T-OCPs have a M1 phenotype. The expression level of iNOS in Ly6C^+^Gr1^-^ cells from T-OCPs was 2-fold higher than in Ly6C^+^Gr1^-^ cells from M-OCPs (p<0.05), but it was 6-fold higher in Ly6C^-^Gr1^-^ cells from T-OCPs than in Ly6C^-^Gr1^-^ cells from M-OCPs probably because the Ly6C^-^Gr1^-^ cells from T-OCPs had also switched to CD11c^+^ M1 cells.

### TNF induction of RelB promotes the differentiation of Ly6C^+^Gr1^-^ and Ly6C^-^Gr1^-^CD11c^+^ macrophages

**Fig 4:**
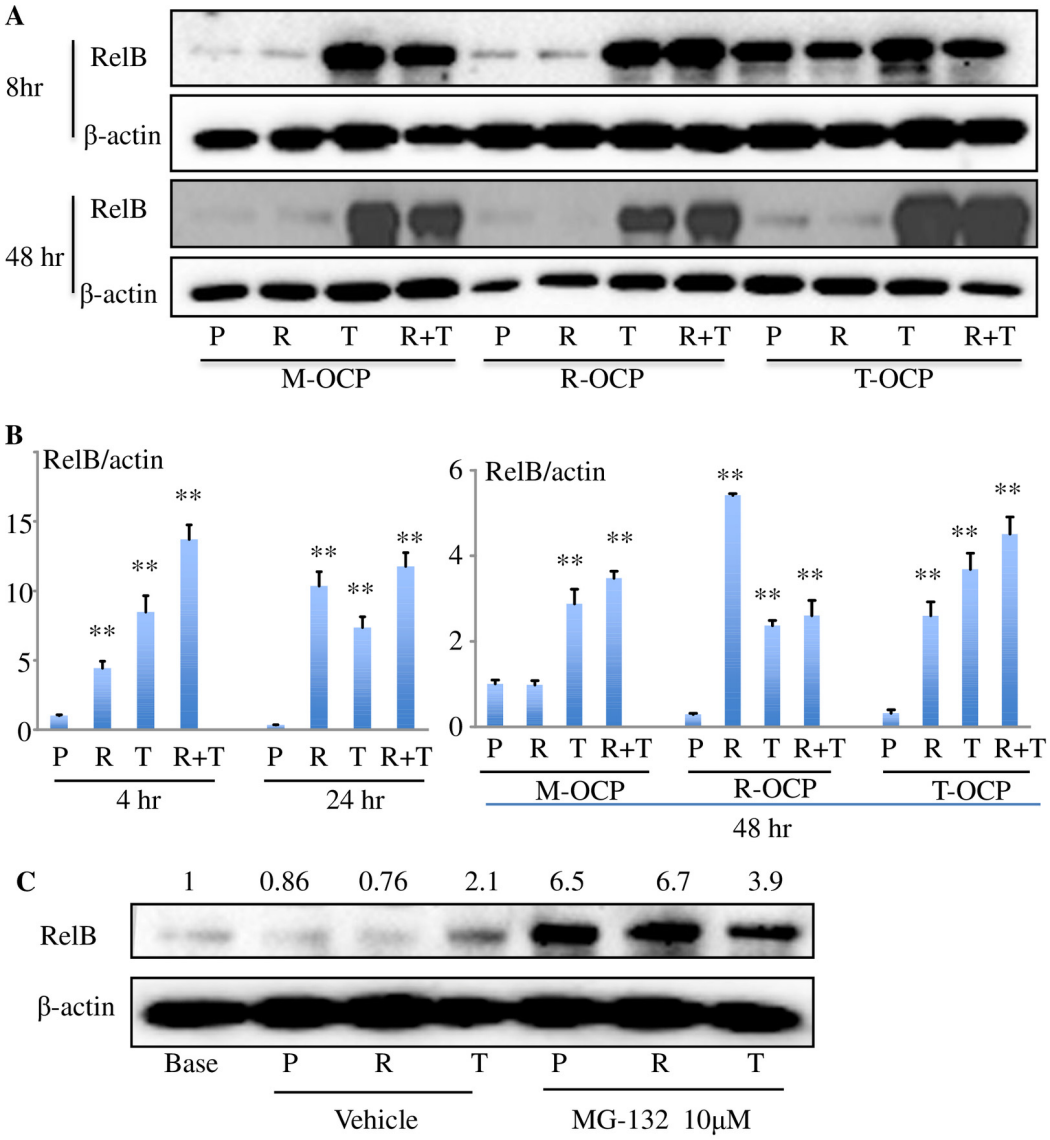
TNF increases expression of RelB mRNA and prevents RelB protein degradation. (A) M-, R-, and T- OCPs generated as in Fig 1B were treated with PBS (P), R, T or R+T for 8 hr or for 48 hr by which time mature OCs had formed. Cell lysates were subjected to Western blot analysis of RelB and β-actin. (B) M-OCPs were treated with P, R, T or R+T for 4 and 24 hr (left panel), or M-, R- and T-OCPs were treated with P, R, T or R+T for 48 hr by which time mature OCs had formed (right panel). Total RNA was extracted to test mRNA expression of NFATc1 normalized to β-actin. **p < 0.01 vs. the respective PBS-treated cells. (C) M-OCPs were serum-starved for 2 hr followed by treatment of P, R or T in the presence of 10 μM MG-132 for 3 hr. Protein levels of RelB and β-actin were tested by Western blot. The data are the band levels measured densitometrically, normalized to β-actin.

RelB is a member of the NF-κB family of transcription factors [34, 35]. OC numbers are normal in the bones of RelB-/- mice, but bone marrow OCPs from RelB-/- mice form fewer OCs than WT OCPs in vitro in response to RANKL [36]. To investigate the role of RelB in TNF-induced OC formation, we first tested RelB protein expression levels in M-, T- and R-OCPs from WT mouse BM cells, generated as in Fig 1B. We then used regular OC-inducing culture medium containing M-CSF and treated the cells with PBS, TNF, RANKL or a combination of TNF and RANKL for an additional 8 or 48 hr (mature OCs being formed at 48 hr). In M- and R-OCPs, RelB protein levels in RANKL-treated cells were similar to or lower than PBS-treated cells, while in contrast, TNF- and TNF+RANKL-treated cells had significantly higher RelB protein levels at both time-points (Fig 4A). After 8 hr treatment, the basal (PBS-treated cells) RelB protein level in T-OCPs was significantly higher than in M- and R-OCPs, while RANKL reduced and TNF increased it slightly (Fig 4A). At 48 hr, RelB protein levels remained low in PBS- and RANKL-treated M- and R-OCPs, while in T-OCPs they dropped to those of M- and R-OCPs and remained high in both TNF- and RANKL+TNF-treated OCPs (Fig 4A).

Increased protein levels can result from increased synthesis and/or decreased degradation. In general, both TNF and RANKL increased RelB mRNA expression (Fig 4B). Compared to PBS, RANKL and TNF increased RelB mRNA expression in M-OCPs by 4.5- and 7.5-fold, respectively, after 4 hr of treatment, and these remained high at 24 hr (Fig 4B). At 48 hr when mature OCs have begun to form, TNF or TNF+RANKL induced 3-fold higher RelB mRNA levels than PBS-treated M-OCPs, but RANKL did not change RelB levels at this time-point. Furthermore, in T- and R-OCPs treated with TNF or RANKL or both in combination RelB mRNA levels were still significantly increased when OCs had formed.

In contrast to the generally increased RelB mRNA, RelB protein levels in RANKL-treated OCPs or mature OCs was lower than or similar to PBS-treated cells (Fig 4A). In many cases, RelB protein levels in RANKL+TNF-treated M-OCPs were slightly lower than in cells treated with TNF alone, suggesting that RANKL may also induce RelB degradation. To test this hypothesis, M-OCPs generated from WT mouse BM cells were serum-starved for 2 hr followed by treatment of vehicle or the proteasome inhibitor, MG-132, for 3 hr. We found that RelB protein levels increased by 7.5- and 9-fold in PBS- and RANKL-treated cells, respectively, in the presence of MG-132, but by only 2-fold in TNF-treated cells (Fig 4C), suggesting that RelB undergoes strong constitutive degradation and that the additional RelB induced by RANKL is also efficiently degraded.

**Fig 5:**
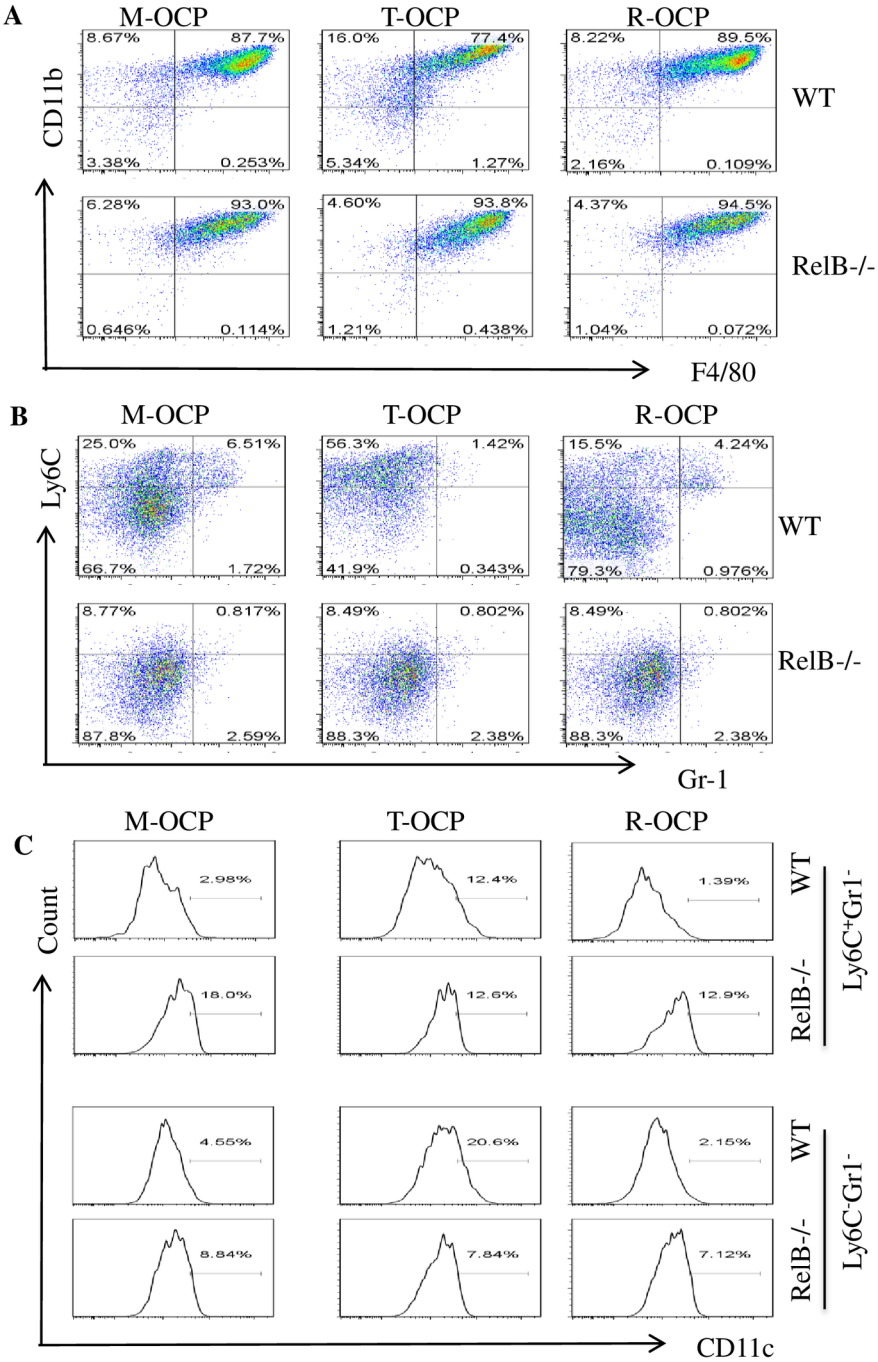
RelB deficiency prevents TNF-induced M1 macrophage differentiation. BMCs from 4-month-old RelB-/- and WT littermate mice were cultured to produce M-, R- and T-OCPs, as in Fig 1B. Cells were subjected to flow cytometry to analyze expression of CD11b and F4/80 (A), Ly6C and Gr1 cells in the CD11b^+^F4/80^+^ population (B), and CD11c^+^ cells in the Ly6C^+^Gr1^-^ and Ly6C^-^Gr1^-^ populations (C), as in Fig 1B. The experiment was repeated twice with similar results.

To determine if TNF induction of Ly6C^+^Gr1^-^ and Ly6C^-^Gr1^-^CD11c^+^ macrophages requires RelB expression, we used RelB-/- mouse BM cells to examine expression of macrophage markers in cytokine-induced OCPs. RelB-/- mice develop multiorgan inflammation [28], and consistent with this, the % CD11b^+^ myeloid cells in freshly isolated RelB-/- BM was higher than in WT cells. For example, CD11b^+^F4/80^+^ cells comprised ~76% of RelB-/- BM cells compared to 45% in WT mice (not shown), and Ly6C^+^ cells were also increased in the CD11b^+^F4/80^+^ population from the RelB-/- mice (not shown). After 3 days of culture, the total percentage of CD11b^+^F4/80^+^ cells from RelB-/- mice was ~93–94% in M-, T- and R-OCPs (Fig 5A). However, TNF did not induce Ly6C^+^Gr1^-^ cells from the RelB-/- CD11b^+^F4/80^+^ population (Fig 5B). Similarly, although the percentage of CD11c^+^ cells in the Ly6C^+^Gr1^-^ and Ly6C^-^Gr1^-^ population of M-OCPs was higher (18% and 8.84%) in RelB-/- cells than in WT cells (2.98% and 4.55%) (Fig 5C), the frequency of CD11c^+^ cells did not change (~12%) in Ly6C^+^Gr1^-^, but was reduced to 7.84% in Ly6C^-^Gr1^-^ cells from RelB-/- T-OCPs compared to their respective WT T-OCPs (12.4% and 20.6%). These data suggest that RelB is required for TNF induction of Ly6C^+^Gr1^-^ and Ly6C^-^Gr1^-^CD11c^+^ cells and that the increase in CD11c^+^ cells seen in RelB-/- mice in vivo is independent of TNF.

### TNF inhibits RANKL-induced osteoclastogenesis from OCPs primed by M-CSF alone, but not by RANKL and TNF

**Fig 6:**
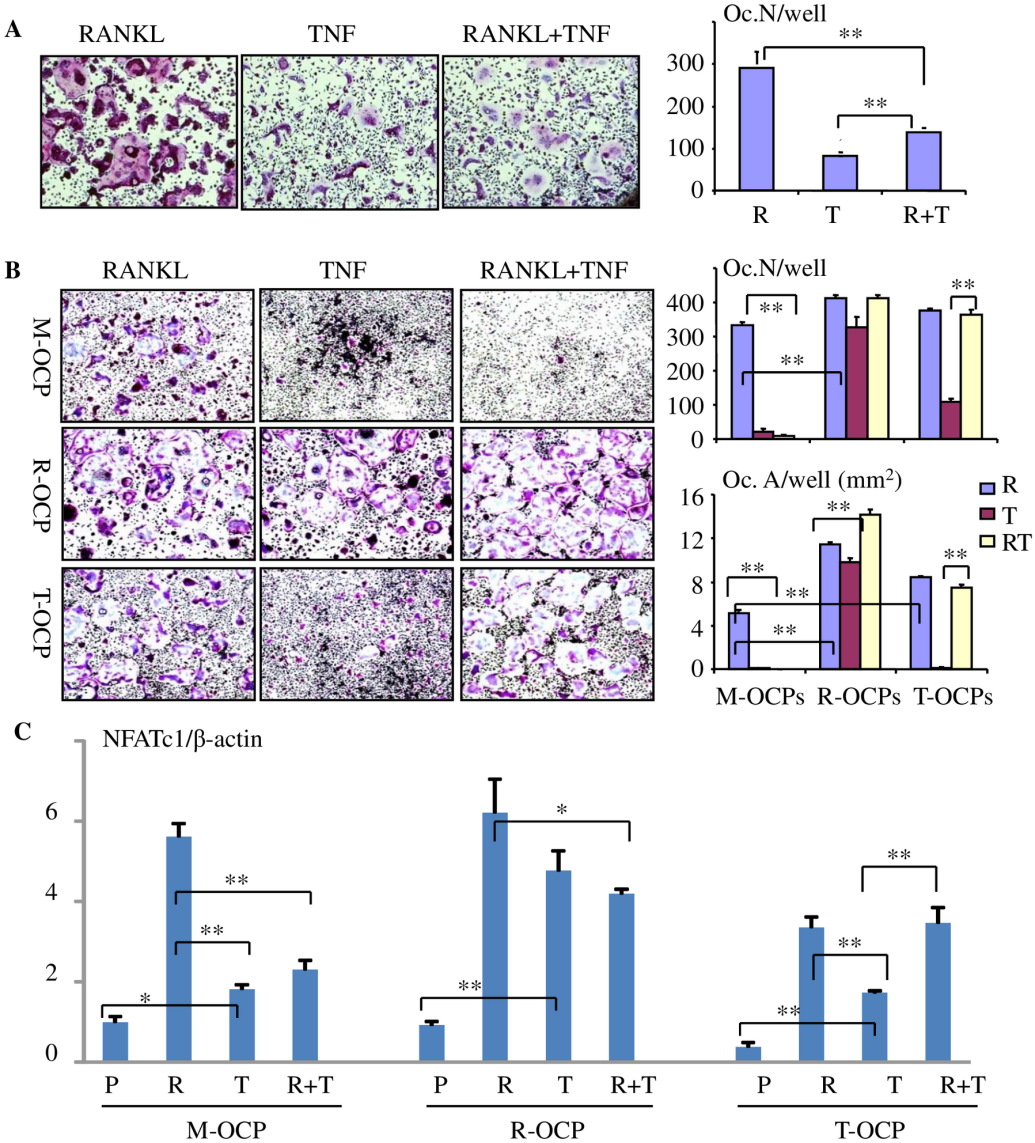
TNF inhibits RANKL-induced osteoclastogenesis from M-OCPs, but not from T-OCPs. (A) BMCs (1x10^5^ per well) from 2-month-old C57Bl6 mice were cultured for M-OCPs in 96-well plates for 2 days, as in Fig 1B. R, T or both were added to the cultures for an additional 2.5 days to generate mature OCs. TRAP staining was performed to evaluate OC numbers, 4 wells per group (*p<0.05, **p<0.01). (B)&(C) BMCs (1x10^5^ per well) from 3-month-old C57Bl6 mice were cultured for M-, R- and T-OCPs in 96-well plates for 2 days. RANKL, TNF or both were added for an additional 2 days in the presence of M-CSF to generate mature OCs. (B) The cells were fixed with 10% neutral buffered formalin for 10 min and TRAP staining was performed to evaluate OC numbers (N) and area (A), 4 wells per group (*p<0.05, **p<0.01). (C) Total RNA was extracted from mature OCs generated from M-, R- and T-OCPs treated with the above cytokines for 48 hr. NFATc1 mRNA expression was tested by real-time PCR normalized to β-actin, **p<0.01. The experiment was repeated twice with similar results.

TNF or RANKL alone induces terminal differentiation of OCPs primed by M-CSF [13, 37]. However, TNF induces significantly fewer osteoclasts than RANKL [16]. TNF increases levels of TNF receptor-associated factor 3 (TRAF3) in OCPs and this induces degradation of NF-κB-inducing kinase (NIK) leading to increased cytoplasmic levels of the inhibitory NF-κB protein, p100, and reduced RANKL- and TNF-induced OCP differentiation [13]. However, TNF also promotes RANKL expression by osteoblastic and other cells to enhance OC formation [20, 21]. To further investigate the conditions in which TNF stimulates or inhibits RANKL-induced OC formation, we cultured BM cells with M-CSF alone or in combination with RANKL or TNF for 2 days to generate M-, R- and T-OCPs, as in Fig 1B. The culture medium was replaced with freshly made medium containing M-CSF and the cells were then treated with TNF, RANKL or RANKL+TNF for an additional 48–60 hr to generate mature OCs. We found that TNF alone induced relatively small numbers of OCs and significantly inhibited RANKL-induced OC formation from M-OCPs (Fig 6A). TNF induced fewer OCs here than in our earlier reports [13, 16] because we stopped these experiments one day earlier to examine early events in OC formation (Fig 6B). In contrast, the numbers and area of OCs induced by TNF from R-OCPs almost matched those induced by RANKL (Fig 6B). The area of OCs induced by TNF+RANKL from R-OCPs was larger than that induced by RANKL alone (p< 0.01, but there was no difference in OC numbers (Fig 6B). Furthermore, RANKL induced more OCs (number and area) from R-OCPs than from M-OCPs (Fig 6B). Although the numbers of RANKL-induced OCs from T-OCPs were similar to those from M-OCPs, the total area of RANKL-induced OCs from T-OCPs was larger than that from M-OCPs (p<0.01), consistent with enhanced fusion. In addition, TNF did not inhibit RANKL-induced OC formation from T-OCPs, the number and area of RANKL+TNF-induced OCs being similar to those induced by RANKL alone (Fig 6B).

To further investigate the conditions in which TNF stimulates or inhibits RANKL-induced OC formation, we next tested mRNA expression levels of NFATc1, the master gene controlling terminal OC differentiation and maturation [38, 39], by real-time PCR. In general, the NFATc1 mRNA expression level matched the number of OCs (Fig 6C). After 4 hr of treatment, neither RANKL nor TNF changed NFATc1 mRNA expression levels (S2 Fig) in M-OCPs, but after 24 hr, RANKL increased NFATc1 mRNA expression by 13-fold. In contrast TNF increased NFATc1 expression by only 2-fold and significantly inhibited RANKL induction of its expression (S2 Fig). After 48 hr, the expression patterns of NFATc1 in mature OCs from M-OCPs in response to RANKL, TNF or RANKL+TNF (Fig 6C) were very similar to those at 24hr (S2 Fig). In contrast, TNF and RANKL+TNF induced similar levels of NFATc1 mRNA expression as RANKL alone in mature OCs from R-OCPs after 48 hr (Fig 6C). In addition, RANKL increased NFATc1 mRNA levels in OCs from T-OCPs significantly more than TNF (Fig 6C). However, the expression level of NFATc1 in OCs induced by RANKL from T-OCPs was only about half of that from M- or R-OCPs (Fig 6C). This may be due to the low basal expression level of NFATc1 in T-OCPs (PBS-treated cells). Of note, TNF did not inhibit RANKL-induced NFATc1 expression in T-OCPs (Fig 6C).

### Biphasic effect of RelB on OC formation

**Fig 7:**
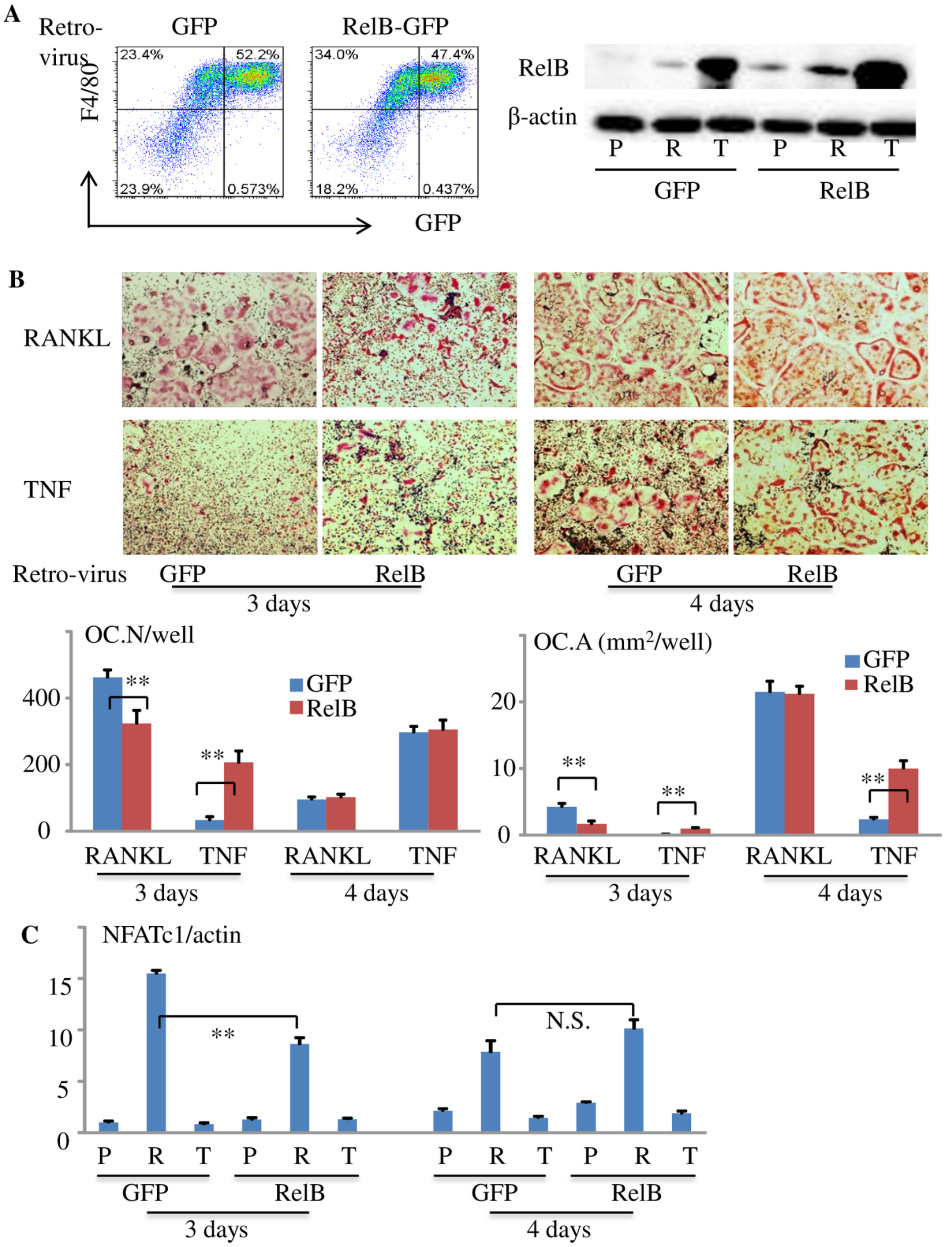
Over-expression of RelB inhibits RANKL-, but enhances TNF-induced OC differentiation. (A) BMCs from 3-month-old C57Bl6 mice were cultured with M-CSF for 2 days followed by treatment of ¼ volume of pMX-GFP or pMX-GFP-RelB retroviral supernatant in the presence of 2 ng/ml of polybrene for 3 days. GFP^+^F4/80^+^ cells were analyzed by flow cytometry (left panel) and RelB protein levels in GFP or RelB expressing cells that had been treated with P, R or T for 8 hr were tested by Western blot (right panel). (B) M-OCPs were infected with pMX-GFP or pMX-GFP-RelB retrovirus as above. After 24 hr of infection, the cells were treated with RANKL or TNF for 3 days or 4 days in the presence of M-CSF when mature OCs were observed under inverted microscopy. TRAP staining was performed to evaluate OC numbers and area, 4 wells per group, *p< 0.05, **p< 0.01. (C) M-OCPs were infected with GFP or GFP-RelB retrovirus and cultured with P, R or T for 3 or 4 days as above (B). Total RNA was extracted from these cells using Trizol reagent, and mRNA expression of NFATc1 normalized to β-actin was tested by real-time PCR. *p< 0.05, **p< 0.01 vs. GFP. The in vitro experiment was repeated twice with similar results.

The precise role of RelB in OC differentiation remains incompletely understood. For example, RANKL-induced OC formation from RelB-/- precursors is impaired in vitro, but the basal OC numbers in RelB-/- mice in vivo are normal [36]. To further investigate the role of RelB in OCP differentiation and OC formation, we over-expressed RelB in WT BM cells using a RelB retrovirus. GFP protein expression in pMX-GFP- and pMX-GFP-RelB retrovirus-infected cells analyzed by flow cytometry and Western blot confirmed RelB over-expression (Fig 7A). We treated the infected cells with RANKL or TNF for 3 or 4 days and found that over-expression of RelB significantly decreased RANKL- but increased TNF-induced OC formation (number and area) after 3 days (Fig 7B). In contrast, after 4 days, the number and area of RANKL-induced OCs were similar in GFP- and RelB-overexpressing cells, reflecting a plateau in OC formation, while OC area induced by TNF from RelB-overexpressing cells was still significantly larger than that from GFP-overexpressing cells, although OC numbers were similar (Fig 7B). We also tested NFATc1 mRNA expression in control and RelB retrovirus-infected M-OCPs treated with TNF or RANKL for 3 or 4 days. We found that TNF did not affect NFATc1 mRNA expression levels in either GFP- or RelB-infected cells. However, RANKL-induced NFATc1 mRNA levels in RelB overexpressing cells were significantly reduced compared to GFP-infected cells after 3 days, but not after 4 days, probably because mature OCs begin to die after 3–4 days (Fig 7C).

## Discussion

M1 and M2 macrophages are linked to T helper 1 (TH1)- and TH2-type immune responses, respectively [33]. M1 macrophages mediate inflammatory responses to a variety of bacterial, protozoal and viral infections and produce many inflammatory cytokines, including TNF, IL-18, IL-12 and IL-23, which mediate immune reactions in several chronic inflammatory and autoimmune diseases, including rheumatoid arthritis, Crohn’s disease, multiple sclerosis and autoimmune hepatitis [40–42]. M2 macrophages, in contrast, inhibit the production of a wide variety of pro-inflammatory mediators, such as IL-10, and regulate wound healing [43]. Thus, targeted depletion of M1 and boosting the activities of M2 macrophages are emerging as an attractive combined therapeutic strategy for autoimmune diseases [44–46]. Better understanding of the mechanisms that regulate M1/M2 differentiation should improve these therapeutic approaches and could lead to reduced joint destruction in inflammatory arthritides.

Granulocyte macrophage-colony stimulating factor (GM-CSF) drives myeloid progenitor differentiation into granulocytes and M1 monocyte/macrophages with a pro-inflammatory cytokine profile (e.g. TNF and IL-23 expression) and also into cells with dendritic cell (DC) properties, and thus it is often employed in studies of DC development and function [47, 48]. However, GM-CSF is not critical for macrophage development since mice lacking GM-CSF do not have notable defects in tissue macrophages [49]. In contrast, targeted ablation of M-CSF or its receptor causes severe depletion of macrophages in many tissues associated with failure of osteoclast formation and osteopetrosis, indicating that M-CSF plays a major role in the generation of macrophages [24].

Macrophages induced in response to M-CSF alone have an anti-inflammatory cytokine profile (e.g. IL-10 expression) and are similar to M2 macrophages [50]. In general, M2 macrophages switch to a M1 phenotype in response to IFN-γ and LPS and secrete large amounts of cytokines involved in autoimmune responses [22, 24, 33, 51]. We previously reported that TNF increases CD11b^+^Gr-1^-/lo^ OCP numbers by stimulating expression of the M-CSF receptor, c-fms [26], which has important roles in OCP proliferation [52], OC formation and survival [53]. However, it was not known if these are M1 or M2 macrophages or if the positive or negative regulatory effects of TNF on OC formation involve modulation of M1/M2 differentiation. Here, we have shown that TNF promotes a switch of M-CSF-induced F4/80^+^CD11b^+^Ly6C^-^Gr1^-^ M2 to F4/80^+^CD11b^+^Ly6C^+^Gr1^-^ and F4/80^+^CD11b^+^Ly6C^-^Gr1^-^CD11c^+^ M1 macrophages based on our findings that: 1) the Ly6C^+^ Gr1^-^ cells comprise 50–63% of CD11b^+^F4/80^+^ from T-OCPs and only 17–25% from M-OCPs; and in contrast, Ly6C^-^Gr1^-^ cells comprise 34–47% of the CD11b^+^F4/80^+^ T-OCPs compared to 67–78% of these cells from M-OCPs (Figs 1B, 2A, 3A and 5B); 2) CD11c^+^ cells in both Ly6C^+^Gr1^-^ and Ly6C^-^Gr1^-^ populations from T-OCPs are also increased compared to the respective populations from M-OCPs (Figs 1B and 5B); and 3) importantly, Ly6C^+^Gr1^-^ cells from both M- and T-OCPs have increased expression of the M1 marker genes, iNOS, TNF, IL-1β and TGFβ1, compared to Ly6C^-^ Gr1^-^ cells. Ly6C^-^Gr1^-^ cells from T-OCPs also have increased expression of iNOS and TGFβ1 compared to those from M-OCPs, and both Ly6C^+^Gr1^-^ and Ly6C^-^Gr1^-^ cells from T-OCPs have decreased expression of the M2 genes, IL-10 and PPAR-γ (Fig 3B).

A switch from a M2 to a M1 phenotype can also change the OC forming potential of these cells. For example, Raw264.7 cells, a murine macrophage cell line that can differentiate into OCs in response to RANKL without the need to add M-CSF, have enhanced OC forming potential when they are induced to a M1 phenotype by IFN-γ and LPS [54]. We found that only Ly6C^+^Gr1^-^ M1 cells, but not Ly6C^-^Gr1^-^ M2 cells in the CD11b^+^F4/80^+^ population from M-OCPs formed OCs in response to RANKL. Thus, CD11b^+^F4/80^+^Ly6C^+^Gr1^-^ M1 macrophages are authentic OCPs. However, both Ly6C^+^Gr1^-^ and Ly6C^-^Gr1^-^ cells primed by TNF have significantly enhanced OC forming potential, and both cell populations have increased expression of the M1 surface marker, CD11c, and express the M1 effector molecules, iNOS, TGFβ1 and IL-1β, suggesting that these cells have been switched to a M1 phenotype. In addition, TNF-primed Ly6C^+/-^Gr1^+^CD11b^+^F4/80^+^ cells form OCs in response to RANKL, although the numbers are small. This population could be similar to our previously identified CD11b^+^Gr1^+/-^ OCPs in TNF-Tg mice [26]. With all of these features, it is not surprising that TNF did not inhibit RANKL-induced OC formation from TNF-primed OCPs. Robust induction of OC formation by TNF from RANKL-primed OCPs probably reflects the fact that these cells are already well along the OC differentiation process since NFATc1 expression was significantly increased within 24 hours of RANKL treatment [16].

In general, the transcription factor, PU.1, controls the global macrophage-specific enhancer repertoire, irrespective of polarization [55]. In response to M1-inducing stimuli, transcription factors, signal transducer and activator of transcription 1 (STAT1) and interferon-regulatory factor 5 (IRF5) are activated to polarize M1 macrophages [55], while factors that activate STAT6, IRF4 and peroxisome proliferator activated receptor-γ (PPAR-γ) control the polarization of M2 macrophages [55]. NF-κB p50 and p65 have been identified as regulators of macrophage polarization and cytokine production [56, 57]. p50 is considered as a key component in the orchestration of M2-driven inflammatory reactions. It inhibits M1-polarization and IFN-β production, and p50-deficient mice show exacerbated M1-driven inflammation and defective capacity to mount allergy-driven M2-polarized inflammatory reactions [57]. Similarly, transfection of p50 siRNA into M2-like macrophages resulted in a significant decrease in expression of the M2 marker, IL-10, and increased production of the M1 markers, IL-12, TNF-α and IL-6 [58]. Hyperacetylated p65 and increased NF-κB binding activity in bone marrow cells with targeted deletion of mammalian sirtuin member 1 (SIRT1) in myeloid cells resulted in increased M1 polarization, migration, and pro-inflammatory cytokine production, suggesting that p65 also plays an important role in M1 activation [56]. Here, we report a novel mechanism by which TNF induction of M1 macrophages is through NF-κB RelB, based on our findings that TNF significantly increased RelB protein levels associated with switching of M-CSF-induced Ly6C^-^Gr1^-^CD11c^-^ M2 to Ly6C^+^Gr1^-^ and Ly6C^-^Gr1^-^CD11c^+^ M1 F4/80^+^CD11b^+^ macrophages, while TNF induction of Ly6C^+^Gr1^-^ and Ly6C^-^Gr1^-^CD11c^+^ M1 macrophages does not occur in RelB-/- bone marrow cells (Fig 5).

The role of RelB in OC differentiation is also poorly understood. OC numbers in the bones of RelB-/- mice are normal in vivo, while RANKL-induced OC formation from RelB-/- myeloid progenitors is impaired in vitro, and cancer-induced osteolysis is reduced in RelB-/- mice in vivo [26]. These findings suggest that RelB is not required for basal OC formation, but appears to play a positive role in the enhanced osteoclastogenesis in pathologic conditions. Contrary to the reported positive role for RelB in OC differentiation [26], we found that RelB itself functions as a transcriptional repressor of NFATc1 to inhibit terminal OC differentiation based on our findings that: 1) TNF inhibits RANKL-induced OC formation and NFATc1 mRNA expression in M-CSF-primed OCPs; and 2) over-expression of RelB significantly inhibits RANKL-induced OC formation and NFATc1 mRNA expression. These are consistent with the reports that RelB acts as a transcriptional repressor of inflammatory mediators by forming an inactive complex with RelA [59–62]. TNF induction of RelB to inhibit NFATc1 activation could explain why TNF alone can significantly increase OCPs numbers through M2 to M1 switching, while having very limited ability to induce terminal OCP differentiation into OCs. In contrast, RANKL strikingly increases OC formation from TNF-primed OCPs compared to OCPs induced by M-CSF alone since it can efficiently degrade RelB protein, resulting in sustained activation of NFATc1 to promote OC formation. Although over-expression of RelB can significantly enhance TNF-induced OC formation, this effect is still lower than that of RANKL since it peaks one day later than that of RANKL. Thus, the ability of TNF alone to induce terminal OC differentiation in physiological and pathologic conditions is limited.

In summary, our findings provide further evidence of positive and negative effects of TNF on OC formation through its induction of RelB. TNF induction of RelB in OCPs limits OC differentiation in the absence of other stimulators and it also directly limits RANKL-induced OC formation by inhibiting NFATc1 activation. However, TNF-induced RelB also directly mediates terminal OC differentiation independently of NFATc1. Our findings show that the dominant role of TNF is to expand the pool of OCPs with enhanced OC forming potential by switching the differentiation of M-CSF-induced M2 resident to M1 inflammatory macrophages. Thus, strategies to degrade RelB could reverse the differentiation of TNF-induced M1 to M2 macrophages and would represent a novel therapeutic approach for inflammatory arthritides.

## Supporting Information

S1 Fig TNF-induced OCPs have reduced frequency of CD11b^+^F4/80^+^ macrophages. (TIFF)

S2 Fig TNF inhibits RANKL-induced NFATc1 mRNA expression. (TIFF)
