# Supplementary figures and images for: TNF Induction of NF-κB RelB Enhances RANKL-Induced Osteoclastogenesis by Promoting Inflammatory Macrophage Differentiation but also Limits It through Suppression of NFATc1 Expression
Source: PLoS One. 2015 Aug 19;10(8):e0135728. doi: 10.1371/journal.pone.0135728 (PMC4545392; doi:10.1371/journal.pone.0135728)

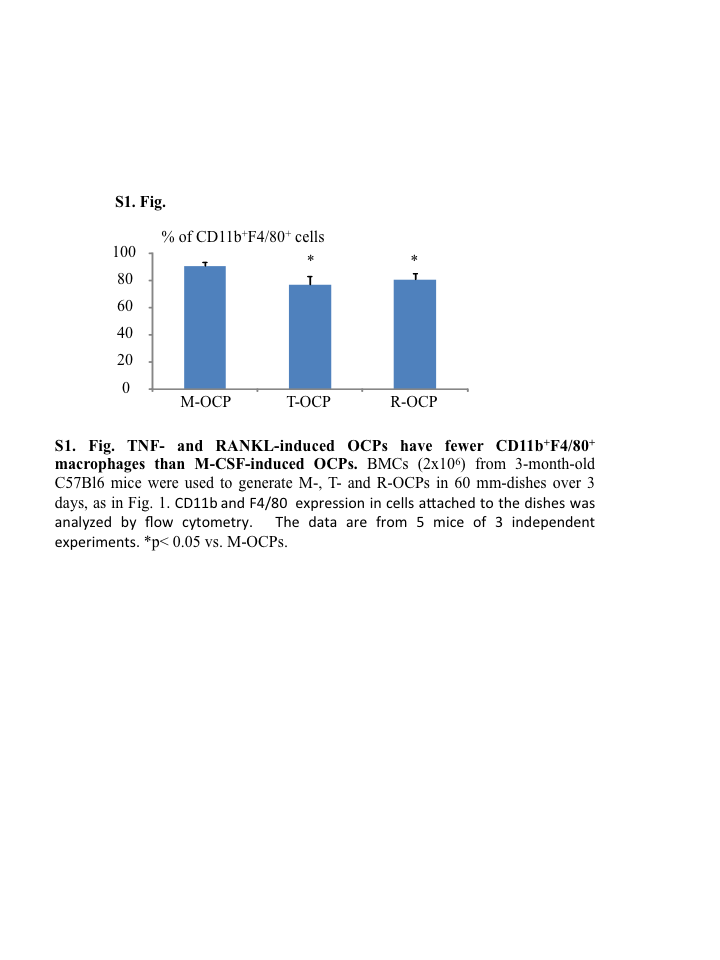

Supplement: S1 Fig — (TIFF) [file pone.0135728.s001.tiff]

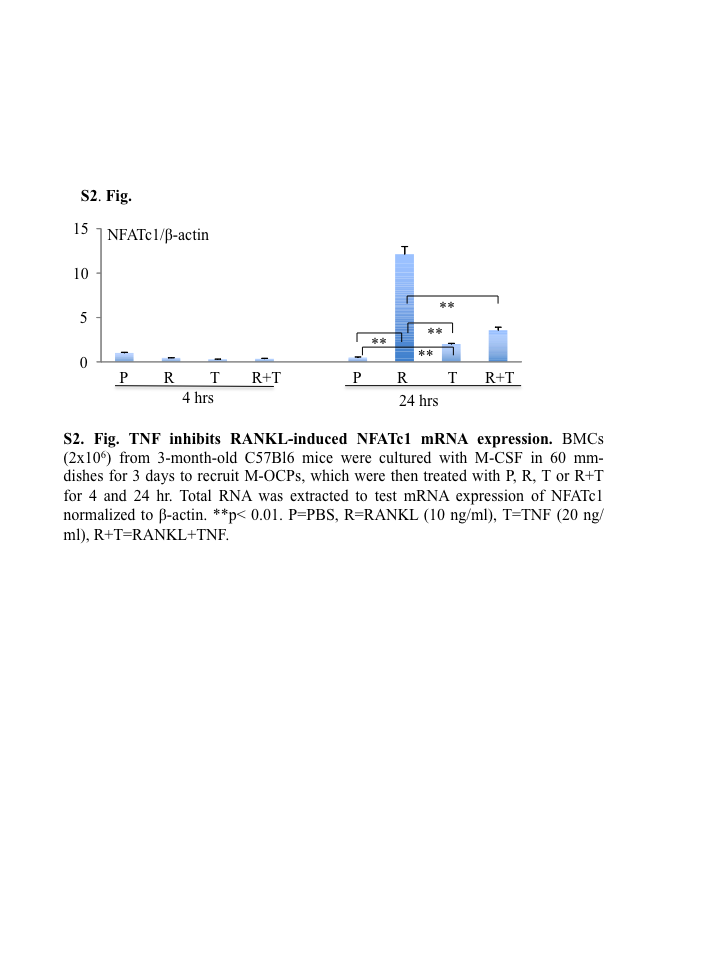

Supplement: S2 Fig — (TIFF) [file pone.0135728.s002.tiff]
